# Graph-based epidemic modeling of West Nile Virus: Forecasting and containment

**DOI:** 10.12688/f1000research.169601.1

**Published:** 2025-09-10

**Authors:** Francesco Branda, Mohamed Mustaf Ahmed, Annamaria Defilippo, Ugo Lomoio, Barbara Puccio, Massimo Ciccozzi, Fabio Scarpa, Pierangelo Veltri, Pietro Hiram Guzzi

**Affiliations:** 1 Unit of Medical Statistics and Molecular Epidemiology,, University Campus Bio-Medico of Rome, Rome, Italy; 2 Genomics, AI, Bioinformatics, Infectious Diseases, Epidemiology Group (GABIE), Rome, Italy; 3 Faculty of Medicine and Health Sciences, SIMAD University, Mogadishu, Banaadir, Somalia; 4 Department of Surgical and Medical Sciences,, Magna Graecia University of Catanzaro, Catanzaro, Italy; 5 Department of Biomedical Sciences, University of Sassari, Sassari, Italy; 6 Department of Computer, Modeling, Electronics and System Engineering, University of Calabria, Rende, Italy

**Keywords:** West Nile Virus, Vector-borne diseases, Transmission dynamics, Decision-support platform, Compartmental models, Ecological interactions

## Abstract

The increasing prevalence of vector-borne diseases like West Nile virus (WNV) highlights the critical need for predictive modeling tools that can guide public health decision-making, particularly given the absence of effective vaccines. We developed a modular computational framework that simulates and analyzes WNV transmission dynamics through compartmental models capturing the intricate ecological interactions among avian hosts, mosquito vectors, and human populations. Our system integrates epidemiological parameters with customizable intervention mechanisms, facilitating the assessment of scenario-specific mitigation approaches. Distinguishing itself from conventional static models, this framework enables users to model dynamic, time-sensitive interventions including targeted mosquito control and strategic bird population management—the two principal containment strategies currently employed against WNV. Using simulations that reflect realistic outbreak scenarios, we evaluated how varying intervention intensities and implementation timings affect epi- demic progression. Our findings reveal that early implemented, dual-target strategies addressing both vector populations and avian reservoirs can substantially reduce transmission dynamics and minimize human exposure risk. This framework serves as a comprehensive decision-support platform for policymakers and vector control agencies, delivering mechanistic insights into the effectiveness of non-pharmaceutical interventions against zoonotic pathogens within complex ecological systems. The tool’s modular design and scenario-testing capabilities make it particularly valuable for proactive outbreak preparedness and evidence-based intervention planning.

## Introduction

**Figure 1.**
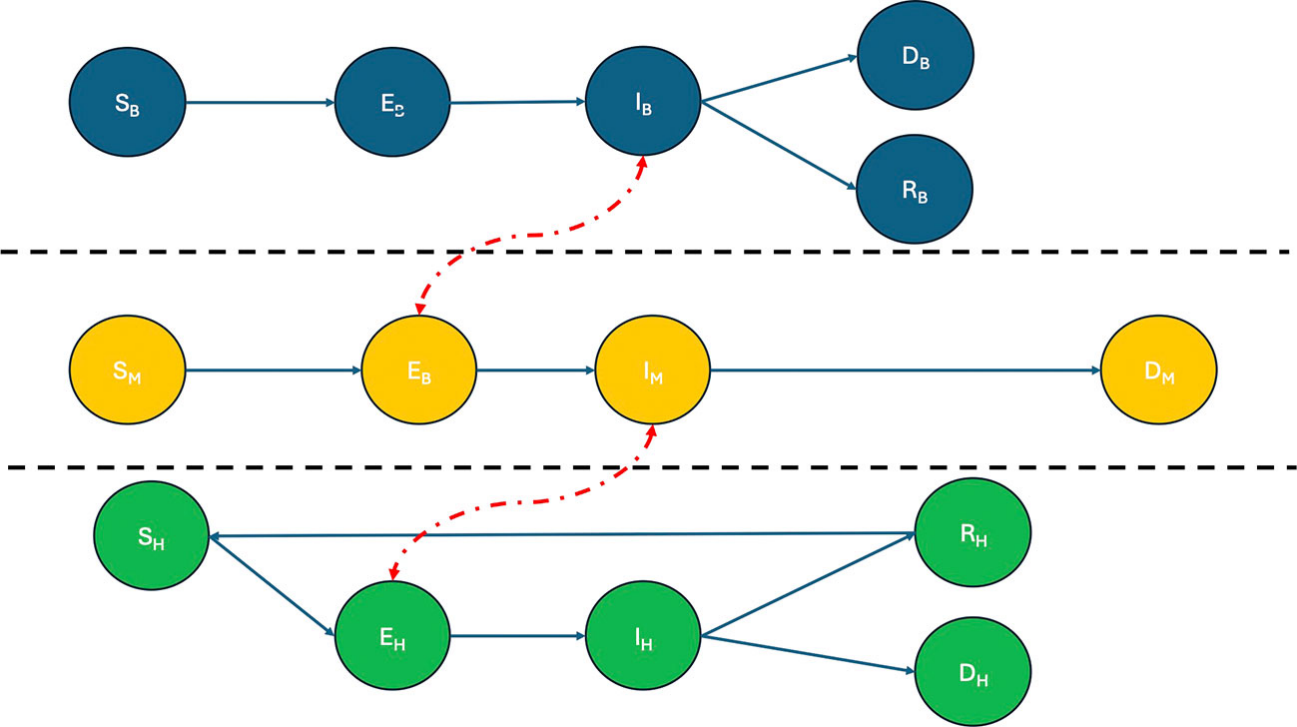
Graph-based SEIRD model for WNV transmission. The figure illustrates the interaction between three epidemiological submodels—birds (top), mosquitoes (center), and humans (bottom)—each represented by a SEIRD compartmental structure. Directed edges between compartments represent progression between epidemiological states (Susceptible, Exposed, Infectious, Recovered, and Dead), while horizontal arrows between populations denote inter-species transmission routes. Specifically, mosquitoes acquire infection from infectious birds ( $I_{b}$ ) and transmit the virus to susceptible humans ( $S_{h}$ ). No direct human-to-human or bird-to-bird transmission occurs. This framework enables the simulation of WNV outbreak dynamics and the assessment of containment strategies such as targeted mosquito population reduction.

The experience of the COVID-19 pandemic has underscored the necessity of complex, adaptive strategies in public health to effectively manage the spread of infectious diseases. ^1^ While COVID-19 triggered global attention, similar computational approaches are equally critical for tackling emerging vector-borne diseases such as West Nile Virus (WNV), whose patterns of transmission and intervention requirements differ substantially from classical airborne viruses. ^2^ In this complex landscape, computational models grounded in mathematical epidemiology and data science ^3^ offer vital tools to simulate, anticipate, and intervene in the dynamics of WNV outbreaks. Unlike diseases where vaccination serves as the main barrier to contagion, WNV presents a distinctive challenge due to the absence of a human vaccine. Instead, effective response hinges on environmental interventions such as mosquito population suppression and limiting avian reservoirs, which can act as amplification hosts for the virus. ^4, 5^ Because WNV transmission involves complex ecological interactions among mosquitoes, birds, and humans, classical compartmental models alone are insufficient. Network-based models provide a more realistic framework by capturing the spatial and contact heterogeneity inherent in vector-host interactions. These models simulate localized dynamics of transmission and allow for the exploration of targeted control strategies, such as geographically selective mosquito eradication or culling of infected bird populations, in order to reduce the risk of human infection. ^6– 9^ This study proposes a simulation-based framework that integrates compartmental disease dynamics with contact-based network representations of WNV spread, as summarized in Figure 1. The framework allows for dynamic updates at the level of individuals or environmental agents, enabling scenario testing under multiple ecological and demographic conditions. It also allows interventions such as the use of larvicides, spraying or habitat destruction, targeted according to simulated risk zones or connectivity measures derived from the network structure.

We focus our analysis on how targeted ecological interventions—rather than mass action or uniform control—can contain or mitigate WNV outbreaks. Using simulation scenarios, we explore the timing, localization, and intensity of mosquito population suppression and its effect on outbreak size, duration, and mortality. The model reveals how different topologies of contact (e.g., clustered bird populations, heterogeneous mosquito densities) affect transmission, and how intervention effectiveness varies accordingly. Using diverse network models reflecting different ecological and urban configurations, the framework highlights the role of adaptive, location-specific responses to WNV threats. The simulations consistently demonstrate that precision targeting—guided by network insights such as centrality or clustering—can dramatically reduce human exposure to the virus, even in the absence of pharmaceutical interventions. Ultimately, this work shows how modern computational tools can support evidence-based public health planning for vector-borne diseases like WNV, offering a testbed to explore and optimise interventions before their real-world deployment. In the absence of vaccines, such approaches are crucial to achieving timely, efficient, and cost-effective epidemic control.

## Materials and methods

Data on WNV cases in Italy were extracted from weekly bulletins published by the Italian national health authorities, available on the EpiCentro platform ( https://www.epicentro.iss.it/westnile/bollettino). The dataset ^10^ collects detailed information on confirmed cases, classified by host, time period and region of origin. All data have been anonymised and aggregated at an administrative level, ensuring full compliance with current data protection regulations.

The data management and integration process were conducted using the R programming language (version 4.5.1) within the RStudio development environment (version 2025.05.1). The workflow involved a series of steps, starting with the cleaning and preparation of the data using the *dplyr* library, which facilitated the elimination of erroneous or inconsistent values. Next, the standardisation of dates was carried out via the *lubridate* library, to ensure uniform handling of time data. In addition, a process of semantic enrichment of the data was implemented, which involved associating the geographical coordinates of the cases with the respective Italian regions, using ISTAT codes. This enrichment made it possible to add contextual information related to the geographical location of the notifications, improving the capacity for spatial analysis.

After the data preparation step, the proposed model was implemented in Python, taking advantage of several open-source libraries and frameworks that support network analysis, simulation, and modular experimentation. At the core of the implementation lies the NetworkX library, ^11^ which was used for the creation, manipulation, and analysis of graph structures. NetworkX provides a flexible and well-documented API that facilitated the representation of complex networks, as well as the computation of key topological properties required for both inference and evaluation.

To simulate network evolution and generate synthetic data reflecting realistic structural patterns, we employed the model introduced by Menczer and Fortunato, ^12^ which offers a principled framework for modeling dynamic and heterogeneous networks. This simulation model allowed us to create controlled experimental conditions for assessing the robustness and generalizability of our method across different types of network topologies and growth dynamics.

The overall architecture and experimentation pipeline were structured using the ExDiff framework, an extensible platform designed for differential and explainable network inference. ^13^ ExDiff provided a modular environment for integrating multiple components—including data preprocessing, inference algorithms, and anomaly detection strategies—while enabling comparative benchmarking under consistent experimental protocols. Its plug-and-play design was essential for evaluating different algorithmic combinations and integration strategies within a unified framework.

All simulations and experiments were conducted using the Google Colab platform ( https://colab.research.google.com/), which offered a scalable and reproducible computational environment equipped with GPU acceleration and cloud-based resources. The use of Google Colab also facilitated collaboration and rapid prototyping, particularly during iterative development and evaluation phases.

The model was evaluated according to a multi-step protocol designed to assess both detection performance and integration efficiency. Simulation was carried out by adoping a network with 1,000 nodes, and a Stochastic Block Model Structure, in which we modelled three communities (Birds, Mosquitos and Human), with a probability of contact within the community p _1_=0.8 and a probability of contacts between the communities p _2_=0.2.

## West Nile diffusion model

WNV circulates within a complex ecological network mainly involving mosquitoes of the *Culex* genus and birds, which act as major reservoirs and amplifiers of the viral load. ^14^ The virus can spillover to incidental hosts, such as humans and horses, leading to a range of clinical manifestations from asymptomatic infection to severe neuroinvasive disease, including meningitis and encephalitis. ^15^ Although these incidental hosts do not contribute substantially to transmission, the health impact of WNV episodes remains significant. Transmission dynamics are driven by a confluence of environmental and ecological factors. Mosquito abundance-one of the strongest predictors of WNV risk-is influenced by temperature and rainfall patterns that directly modulate vector capacity and the rate of viral replication within the vector. ^16^ In addition, the seasonal migration of birds influences the spatial and temporal availability of susceptible reservoir hosts, creating transient hotspots of viral amplification. These ecological variables, combined with human behaviour and urbanisation patterns, shape the landscape of WNV transmission and contribute to its spatial and temporal heterogeneity. Effective management of WNV requires an integrated understanding of the interactions between arthropod vectors, avian reservoirs and environmental modulators of risk. ^17, 18^ Multidisciplinary approaches combining entomological surveillance, ecological modelling and computational simulations are therefore essential to anticipate outbreak trajectories and design tailored vector control strategies. As no human vaccine currently exists, interventions must focus on suppressing mosquito populations and disrupting vector-host-outbreak contact chains that critically depend on the predictive insights offered by dynamic models. To capture the epidemiological dynamics of WNV, we adopt an extended SEIRD (Susceptible-Exposed-Infectious-Recovered-Dead) ^19, 20^ compartmental model that incorporates multiple host populations-birds, mosquitoes and humans-each with distinct biological and epidemiological roles.

**Figure 2.**
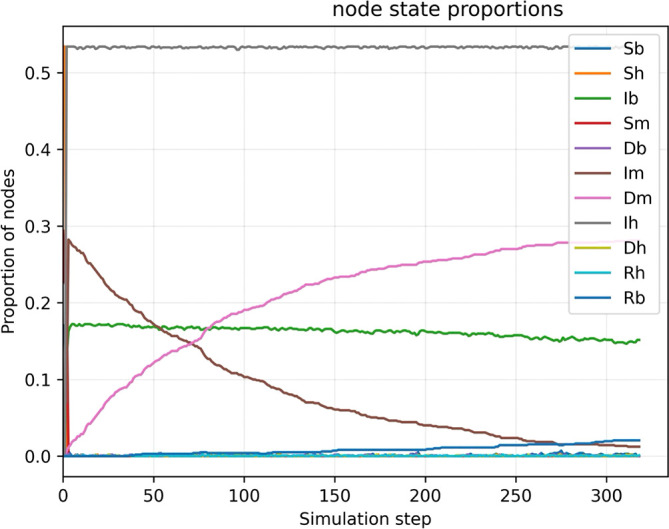
Baseline simulation. Figure shows that the fraction of $I_{h}$ rapidly goes to 50%, the fraction of $I_{m}$ decreases after the begin of the outbreak. It is important to note that the fraction of $I_{b}\;$ remains still high since the transmission within the bird community is autosustained.

The interaction between these populations is encoded within a heterogeneous contact graph that is dynamic in time. A graphical representation of the structure of the model is shown in Figure 2. Birds act as the main amplifying hosts for WNV, and their dynamics are described via the compartments: Sb (Susceptible) for birds at risk of infection, $E_{b}$ (Exposed) for birds infected by a mosquito but not yet infectious, $I_{b}$ (Infectious) for birds capable of transmitting the virus to mosquitoes, $R_{b}$ (Recovered) for birds that have recovered and acquired immunity, $D_{b}$ (Dead) for birds that succumb to WNV infection. Mosquitoes, which act as vectors of transmission between birds and humans, do not recover from infection, but their life cycle includes mortality from both natural causes and control strategies: $S_{m}$ (Susceptible) for mosquitoes that have not yet acquired the virus, $E_{m}$ (Exposed) for mosquitoes that have bitten an infected bird but are still in the extrinsic incubation period, $I_{m}$ (Infectious) for mosquitoes capable of transmitting WNV, $D_{m}$ (Dead) for mosquitoes that die from natural causes, infection or interventions such as the use of larvicides or adulticides. Humans are considered incidental, end-of-cycle hosts, meaning that they do not contribute significantly to transmission. However, modelling morbidity and mortality is essential to capture health outcomes: $S_{h}$ (Susceptible) for individuals vulnerable to WNV infection, $E_{h}$ (Exposed) for individuals bitten by an infected mosquito and incubating the virus, $I_{h}$ (Infectious) for symptomatic individuals, an essential compartment for tracking the disease burden, $R_{h}$ (Recovered) for individuals who survive infection and acquire immunity, $D_{h}$ (Dead) for individuals who die due to WNV-related complications such as encephalitis or neuroinvasive disease.

SEIRD dynamics are interconnected through a graph-based interaction scheme reflecting biological transmission pathways: the Mosquito-Bird arcs represent the central zoonotic cycle for WNV amplification, while the Mosquito-Human arcs model the incidental spillover from the enzootic cycle to humans. There are no direct transmission arcs between humans or between birds, all transmission is vector-mediated. This modelling strategy enables high-resolution simulation of outbreak dynamics and evaluation of vector control interventions (e.g. mosquito population reduction), which is currently the only effective containment strategy in the absence of a human or avian vaccine. Figure 1 illustrates the multi-host SEIRD model and the direct arcs encoding contact-based interactions that are critical for WNV transmission and control.

## Results

### Case Study 1: Uncontrolled diffusion

Our baseline scenario simulates an uncontrolled WNV outbreak within a densely interconnected ecological network, with no containment intervention. This simulation establishes the basic conditions for the outbreak, characterised by high densities of mosquito vectors and a large population of susceptible birds-factors that favour continuous viral amplification and virus transmission. Human exposure occurs mainly through contact with infected mosquitoes that act as a transmission bridge. In the absence of vector control measures or environmental management, the simulation follows the natural propagation of the virus, which is governed solely by basic biological parameters: the competence of the vector, the incubation periods of the pathogen and the feeding behaviour of mosquitoes.

The basic results, summarized in Figure 2, reveal a rapid and extensive spread of WNV throughout the ecological network, marked by an exponential rise in infections among both mosquito and bird populations during the initial phases of transmission. The high degree of connectivity among bird populations—particularly migratory species that bridge geographically distant communities—enables efficient long-range dissemination of the virus. This structural feature acts as a powerful driver of ecological amplification, sustaining viral circulation across spatially dispersed regions. As the simulation progresses, the fraction of infected birds (IbI_bIb) stabilizes at approximately 30%, a level maintained through self-sustaining intra-species transmission within avian communities. This persistent reservoir of infection in birds serves as a key engine for ongoing transmission. In contrast, the fraction of infected mosquitoes gradually declines to around 10%, a consequence of their short lifespan and limited capacity to sustain prolonged transmission chains. Despite this decline, mosquitoes remain essential vectors for cross-species transmission.

Notably, the fraction of infected humans increases substantially, reaching 50% by the end of the simulation. This sharp rise illustrates the significant spillover risk posed by the interaction of persistent avian reservoirs and mosquito vectors, especially in the absence of effective control measures. Without ecological interventions—such as larvicide application, adult mosquito suppression, or systematic monitoring of bird populations—the epidemic rapidly approaches critical transmission thresholds, saturating network pathways and resulting in a substantial cumulative burden of infection in the human population.

The results highlight the serious public health consequences of passive or delayed WNV response protocols. Natural transmission dynamics, when enhanced by favourable environmental conditions, can rapidly exceed the capacity of the local health system in the absence of timely and geographically targeted interventions. This baseline scenario establishes the essential benchmarks for the evaluation of subsequent simulations, which incorporate active containment strategies, allowing for a quantitative assessment of the effectiveness of interventions - either through the suppression of mosquito populations or through the interruption of transmission cycles between birds and vectors.

### Case Study 2: Simulation of vector control intervention targeting mosquitoes

The containment scenario evaluates the impact of intensive vector control strategies designed to drastically suppress mosquito populations, the critical transmission bridge for WNV. We simulate this strategy by removing significant proportions of mosquito nodes and their transmission links from the contact network, thereby cutting transmission paths between vectors and hosts. This approach simulates comprehensive control programmes, including large-scale larvicide deployment, adulticide spraying campaigns and targeted habitat eradication initiatives. By maintaining the integrity of bird and human population networks, but isolating the vector component, we can directly quantify the effects of mosquito suppression on epidemic trajectories.

**Figure 3.**
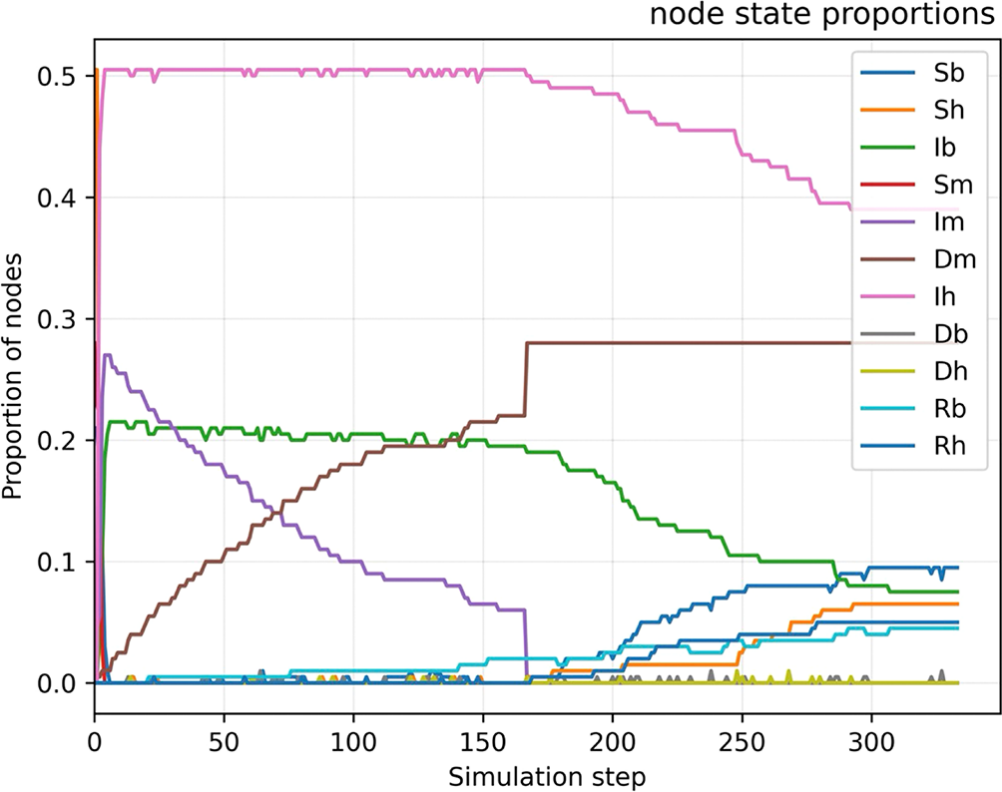
Simulation of intervention to kill mosquitoes. Simulation of WNV transmission dynamics across an ecological network comprising birds, mosquitoes, and humans. In the early phases, the infection spreads rapidly, with bird infections stabilizing at ~30%, mosquito infections peaking and then declining to ~10% due to short lifespans, and human infections rising to ~50%. At simulation step 175, a targeted intervention eliminates all mosquitoes, reducing their infected fraction to 0%. This leads to a rapid drop in human infections while bird infections remain unchanged. The interruption of the transmission bridge effectively halts cross-species spread, highlighting the critical role of vector control.

The results, shown in Figure 3 reveal a rapid and extensive spread of WNV throughout the ecological network, marked by an exponential rise in infections among both mosquito and bird populations during the initial phases of transmission. The high degree of connectivity among bird populations—particularly migratory species that link geographically distant communities—enables efficient long-range dissemination of the virus. This structural feature acts as a powerful driver of ecological amplification, sustaining viral circulation across spatially dispersed regions. As the simulation progresses, the fraction of infected birds stabilizes at approximately 30%, a level maintained through self-sustaining intra-species transmission within avian communities. This persistent reservoir of infection in birds serves as a key engine for ongoing viral propagation. In contrast, the fraction of infected mosquitoes declines gradually to around 10%, reflecting their short lifespan and limited capacity for long-term transmission chains. Nevertheless, mosquitoes remain critical for cross-species transmission, particularly to humans. Notably, the fraction of infected humans rises substantially over time, reaching 50% by the end of the simulation.

This pronounced increase highlights the significant spillover risk posed by the interaction between persistent avian reservoirs and mosquito vectors. In the absence of ecological interventions—such as larvicide application, adult mosquito control, or avian population monitoring—the epidemic rapidly approaches critical transmission thresholds, saturating available pathways and resulting in a high cumulative burden of human infection. To explore potential mitigation strategies, we simulated a targeted intervention at time step 175, in which all mosquitoes were eliminated from the network. This action resulted in an immediate and complete collapse of the mosquito population (with the infected mosquito fraction dropping to 0%) and triggered a sharp decline in human infections, with the fraction of infected birds decreasing rapidly in the following steps. Interestingly, the fraction of infected birds remained stable at 30%, indicating the continued presence of a viral reservoir. However, the removal of the mosquito population effectively disrupted the transmission bridge between birds and humans, halting further spread of the virus across species. This outcome underscores the critical role of vector control in breaking transmission pathways, even in the presence of persistent ecological reservoirs.

These results provide convincing evidence of the effectiveness of intensive mosquito control in suppressing WNV epidemic outbreaks. The drastic reduction of vector density brings the basic reproduction number below the critical threshold of 1, effectively interrupting self-sustaining transmission cycles. Although this scenario represents an idealised outcome, which may prove difficult to realise operationally, it provides a solid theoretical justification for prioritising vector control in WNV response protocols.

The simulation also serves as a performance benchmark for evaluating partial or delayed intervention strategies, highlighting the importance of rapid and geographically coordinated responses to emerging vector transmission signals. This analysis emphasises that effective vector control, if implemented in a comprehensive and timely manner, can be the cornerstone of WNV containment efforts.

## Discussion

The results of this study strongly highlight the potential of network-based computational models to address the spread of WNV under realistic and complex scenarios. Unlike static approaches, the proposed framework enables dynamic simulation of the interactions among reservoir hosts, vectors, and human populations, incorporating targeted ecological interventions such as selective mosquito suppression or strategic management of bird populations. These capabilities are crucial in a context where no approved human vaccine is available, and public health responses must rely on non-pharmaceutical interventions. The two simulated scenarios clearly demonstrate the effectiveness of containment strategies. In the uncontrolled outbreak scenario, the epidemic spreads rapidly through the ecological network, with a surge in infected birds and mosquitoes and a significant increase in human cases. High connectivity among avian populations, especially migratory species, facilitates long-range viral transmission, illustrating how even minimal delays in intervention can lead to saturation of transmission pathways. In contrast, the scenario involving targeted vector suppression shows a substantial reduction in transmission, ultimately breaking the epidemiological chain. The drastic decrease in mosquito density reduces the number of spillover events to humans and prevents the virus from reaching a sustainable transmission level. These findings emphasize the importance of timely, geographically coordinated strategies for WNV control, confirming that well-implemented vector control measures remain one of the most effective tools for responding to vector-borne diseases.

A key role in this context has been played by ArboItaly, ^21^ a platform implemented by GABIE research group ( https://gabie-r.web.app/) for national surveillance network for arboviral diseases in Italy, which provided essential data for the spatial and temporal analysis of WNV transmission. Integration of data from ArboItaly—particularly confirmed cases, and vector distribution—allowed the simulation model to be calibrated at the territorial level and enabled the assessment of intervention impact under realistic conditions. The ArboItaly experience demonstrates how the availability of real-time, high-quality data is now an indispensable tool for effective and timely health decision-making. ^22, 23^ The ability to track evolving risk week by week, through a combination of entomological, virological, and environmental information, allows not only early detection of outbreak signals but also precise tailoring of control measures and optimization of available resources. The continuous flow of reliable, up-to-date information among national institutions, local health authorities, and regional laboratories enhances the responsiveness of the entire health system, enabling a truly One Health approach and, at the same time, promoting transparency through accessible and regularly updated communication tools.

## Data Availability

The static version of the dataset is deposited in Zenodo and accessible at https://zenodo.org/records/8355821. ^24^ To facilitate data reuse and ensure continuous updates, we also provide metadata, R scripts, and a dynamically maintained dataset in a dedicated GitHub repository: https://github.com/fbranda/west-nile . Data are available under the terms of the Creative Commons Attribution 4.0 International license (CC-BY 4.0).
