# Graph-based epidemic modeling of West Nile Virus: Forecasting and containment

**DOI:** 10.12688/f1000research.169601.3

**Published:** 2025-12-19

**Authors:** Francesco Branda, Mohamed Mustaf Ahmed, Annamaria Defilippo, Ugo Lomoio, Barbara Puccio, Massimo Ciccozzi, Fabio Scarpa, Pierangelo Veltri, Pietro Hiram Guzzi

**Affiliations:** 1 Unit of Medical Statistics and Molecular Epidemiology,, University Campus Bio-Medico of Rome, Rome, Italy; 2 Genomics, AI, Bioinformatics, Infectious Diseases, Epidemiology Group (GABIE), Rome, Italy; 3 Faculty of Medicine and Health Sciences, SIMAD University, Mogadishu, Banaadir, Somalia; 4 Department of Surgical and Medical Sciences,, Magna Graecia University of Catanzaro, Catanzaro, Italy; 5 Department of Biomedical Sciences, University of Sassari, Sassari, Italy; 6 Department of Computer, Modeling, Electronics and System Engineering, University of Calabria, Rende, Italy

**Keywords:** West Nile Virus, Vector-borne diseases, Transmission dynamics, Decision-support platform, Compartmental models, Ecological interactions

## Abstract

The increasing prevalence of vector-borne diseases like West Nile virus (WNV) highlights the critical need for predictive modelling tools that can guide public health decision-making, particularly given the absence of effective vaccines. We developed a modular computational framework that simulates and analyses WNV transmission dynamics through compartmental models capturing the intricate ecological interactions among avian hosts, mosquito vectors, and human populations. Our system integrates epidemiological parameters with customizable intervention mechanisms, facilitating the assessment of scenario-specific mitigation approaches. Distinguishing itself from conventional static models, this framework enables users to model dynamic, time-sensitive interventions including targeted mosquito control and strategic bird population management, the two principal containment strategies currently employed against WNV. Using simulations that reflect realistic outbreak scenarios, we evaluated how varying intervention intensities and implementation timings affect epidemic progression. Our findings reveal that early implemented, dual-target strategies addressing both vector populations and avian reservoirs can substantially reduce transmission dynamics and minimize human exposure risk. This framework serves as a comprehensive decision-support platform for policymakers and vector control agencies, delivering mechanistic insights into the effectiveness of non-pharmaceutical interventions against zoonotic pathogens within complex ecological systems. The tool’s modular design and scenario-testing capabilities make it particularly valuable for proactive outbreak preparedness and evidence-based intervention planning.

## Introduction

The experience of the COVID-19 pandemic has underscored the necessity of complex, adaptive strategies in public health to effectively manage the spread of infectious diseases. ^1^ While COVID-19 triggered global attention, similar computational approaches are equally critical for tackling emerging vector-borne diseases such as West Nile Virus (WNV), whose patterns of transmission and intervention requirements differ substantially from classical airborne viruses. ^2^ In this complex landscape, computational models grounded in mathematical epidemiology and data science ^3^ offer vital tools to simulate, anticipate, and intervene in the dynamics of WNV outbreaks. Unlike diseases where vaccination serves as the main barrier to contagion, WNV presents a distinctive challenge due to the absence of a human vaccine. Instead, effective response hinges on environmental interventions such as mosquito population suppression and limiting avian reservoirs, which can act as amplification hosts for the virus. ^4, 5^ Because WNV transmission involves complex ecological interactions among mosquitoes, birds, and humans, classical compartmental models alone are insufficient. Network-based models provide a more realistic framework by capturing the spatial and contact heterogeneity inherent in vector-host interactions. These models simulate localized dynamics of transmission and allow for the exploration of targeted control strategies, such as geographically selective mosquito eradication or culling of infected bird populations, in order to reduce the risk of human infection. ^6– 9^

In Italy, the *ArboItaly* platform, ^10^ developed by the GABIE research group, exemplifies how integrated surveillance systems can provide the high-quality, real-time data required for spatially explicit modelling of WNV. By combining entomological, virological, and environmental information, such infrastructures enable timely calibration of models and support adaptive, evidence-based interventions. ^11, 12^ This highlights the crucial role of surveillance data in bridging computational modelling with actionable public health decision-making.

**Figure 1.**
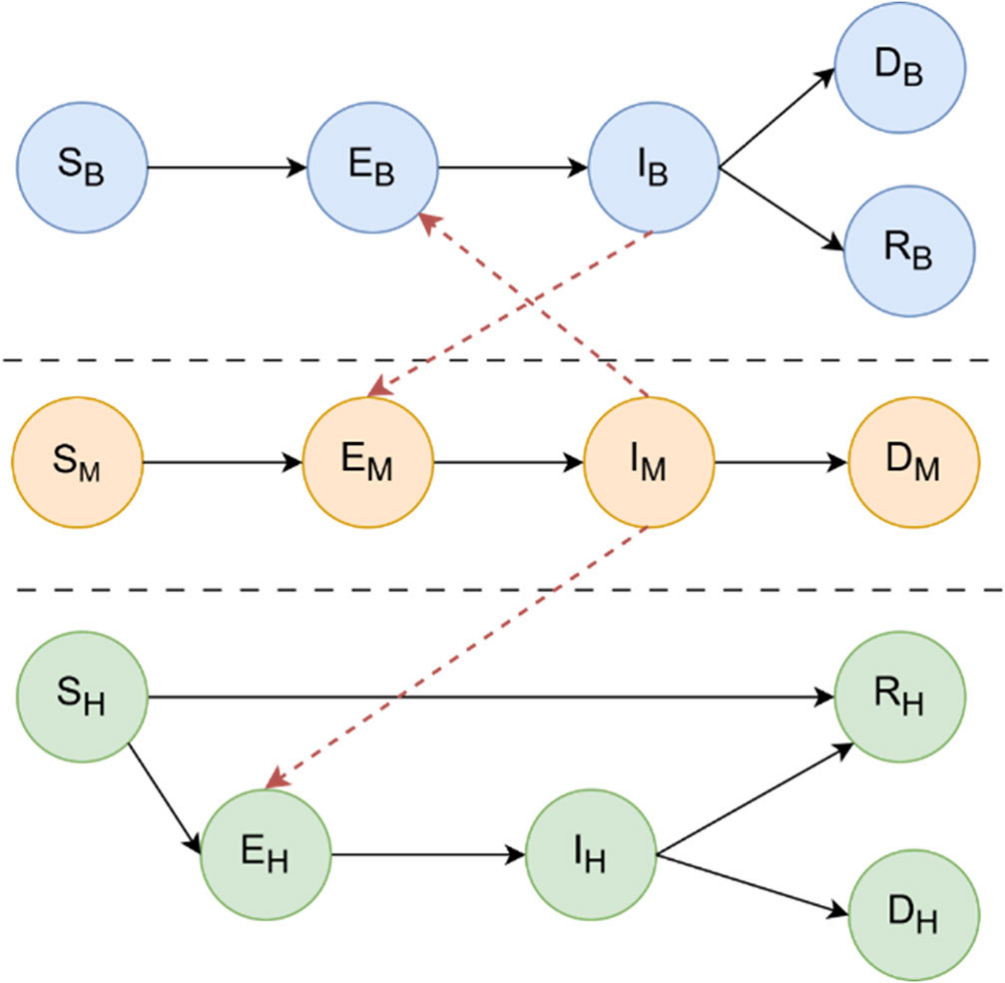
Graph-based SEIRD model for WNV transmission. The figure illustrates the interaction between three epidemiological sub-models: birds (top), mosquitoes (center), and humans (bottom), each represented by a SEIRD compartmental structure. Directed edges between compartments represent progression between epidemiological states (Susceptible, Exposed, Infectious, Recovered, and Dead), while horizontal arrows between populations denote inter-species transmission routes. Specifically, mosquitoes acquire infection from infectious birds ( $I_{B}$ ) and transmit the virus to susceptible humans ( $S_{H}$ ). No direct human-to-human or bird-to-bird transmission occurs. This framework enables the simulation of WNV outbreak dynamics and the assessment of containment strategies such as targeted mosquito population reduction. Additional details on the mathematical equations governing transitions between the compartments are available in section *West Nile diffusion model*.

This study proposes a simulation-based framework that integrates compartmental disease dynamics with contact-based network representations of WNV spread. For this purpose, Figure 1 summarises the interactions between the three SEIRD (Susceptible, Exposed, Infectious, Recovered, and Dead) epidemiological sub-models that drive the compartmental structure. The framework allows for dynamic updates at the level of individuals or environmental agents, enabling scenario testing under multiple ecological and demographic conditions. It also allows interventions such as the use of larvicides, spraying or habitat destruction, targeted according to simulated risk zones or connectivity measures derived from the network structure.

We focus our analysis on how targeted ecological interventions, rather than mass action or uniform control, can contain or mitigate WNV outbreaks. Using simulation scenarios, we explore the effects of interventions on mosquito and bird populations on human infection. The model reveals how different topologies of contact (e.g., clustered bird populations, heterogeneous mosquito densities) affect transmission, and how intervention effectiveness varies accordingly. Allowing the use of diverse network models reflecting different ecological and urban configurations, the framework highlights the role of adaptive, location-specific responses to WNV threats. The simulations consistently demonstrate that precision targeting, guided by network insights, can significantly reduce human exposure to the virus, even without pharmaceutical interventions. Therefore, the primary aim of this paper is to show how a novel, explainable AI framework can uniquely combine compartmental epidemic modelling with Graph Neural Networks (GNNs) and interpretability.

Ultimately, this work demonstrates how modern computational tools can support evidence-based public health planning for vector-borne diseases like WNV, providing a testbed to explore and optimise interventions before real-world deployment, even when the experiments are simulated scenarios. In the absence of vaccines, such approaches are crucial to achieving timely, efficient, and cost-effective epidemic control.

## Related Work

Computational modelling of vector-borne diseases has traditionally relied on compartmental frameworks (e.g., SEIR/SEIRD models) and network-based approaches to capture the complex interplay between hosts, vectors, and environmental factors. In the case of WNV, early studies emphasised statistical and mechanistic models to forecast outbreaks, but recent advances in GNNs have opened new avenues for spatially explicit, data-driven prediction.

Several works have demonstrated the potential of GNNs for WNV forecasting. Tonks et al. ^13^ introduced spatially aware GNN models based on GraphSAGE layers, leveraging mosquito surveillance data from Illinois to predict WNV presence. Their results highlighted the importance of capturing spatial dependence in irregularly sampled geospatial data, showing that GNNs outperform traditional baselines in epidemic forecasting. Similarly, Bonicelli et al. ^14^ applied GNN-based aggregation (specifically a multiadjacency graph attention network) to model spatial circulation patterns of WNV, considering multiple relations, integrating Earth Observation data to account for environmental drivers of transmission (such as the differences in temperature and soil moisture between two sites, along with the geographical distance between them).

Beyond WNV, GNNs have also been applied to other vector-borne diseases. For instance, attention-based GNNs have been employed in dengue severity prediction, improving predictive accuracy and emphasizing crucial clinical indicators through the use of attention mechanisms. ^15^ More broadly, recent reviews emphasize the growing role of GNNs in epidemic modelling, noting their ability to integrate heterogeneous data sources and uncover latent transmission structures. ^16^ Novel architectures such as multi-scale spatiotemporal GNNs (MSGNN) ^17^ and graph attention-based epidemic models ^18^ further demonstrate the adaptability of GNNs to capture long-range dependencies and dynamic disease spread.

At the intersection of mechanistic epidemiology and explainable AI, recent work has explored hybrid models that combine epidemiologically informed neural networks (EINNs) with data-driven learning. ^19^ Similarly, explainable GNN frameworks such as GNN-SubNet ^20^ have been developed to identify influential subnetworks in biomedical contexts, underscoring the importance of interpretability in high-stakes domains like public health. However, despite these advances, no prior study has explicitly integrated compartmental epidemic dynamics with GNN-based representation and explainability modules.

Our work addresses this gap by coupling a SEIRD compartmental structure with a graph-based representation of vector-host-human interactions, enriched by explainability mechanisms that identify influential nodes and transmission pathways. This integration not only advances prior compartmental and GNN-based approaches but also establishes a methodological bridge between mechanistic epidemiology and explainable artificial intelligence, offering novel insights into intervention effectiveness and disease dynamics.

## Materials and Methods

Data on WNV cases in Italy were extracted from weekly bulletins published by the Italian national health authorities, available on the EpiCentro platform ( https://www.epicentro.iss.it/westnile/bollettino). The dataset ^21^ collects detailed information on confirmed cases, classified by host, time period and region of origin. All data have been anonymised and aggregated at an administrative level, ensuring full compliance with current data protection regulations.

The data management and integration process were conducted using the R programming language (version 4.5.1) within the RStudio development environment (version 2025.05.1). The workflow involved a series of steps, starting with the cleaning and preparation of the data using the *dplyr* library, which facilitated the elimination of erroneous or inconsistent values. Next, the standardisation of dates was carried out via the *lubridate* library, to ensure uniform handling of time data. In addition, a process of semantic enrichment of the data was implemented, which involved associating the geographical coordinates of the cases with the respective Italian regions, using ISTAT codes. This enrichment made it possible to add contextual information related to the geographical location of the notifications, improving the capacity for spatial analysis.

Importantly, these data were employed solely for contextual enrichment and qualitative validation of the simulated epidemic trajectories, ensuring that the scenarios reflect realistic outbreak dynamics. Finally, to guarantee biological realism, all key epidemiological and kinetic parameters, including incubation durations, recovery times, disease-induced mortality rates, and inter-species transmission probabilities, were derived exclusively from a systematic review of the established literature on WNV epidemiology and modelling. ^22– 29^ These parameters are summarised in Table 1, which outline the SEIRD model configuration across birds, mosquitoes, and humans, including species-specific transmission rates, incubation periods, and mortality factors.

**Table 1.** Epidemiological and biological parameters used to simulate WNV dynamics across birds, mosquitoes, and humans in SEIRD dynamics. Values include species-specific incubation periods, recovery rates, transmission probabilities, and disease-induced mortality, derived from a systematic review of established literature on WNV epidemiology and modelling.

Parameter	Value	Description
$\beta_{\mathrm{MB}}$	0.30	Transmission Mosquito → Bird
$\beta_{\mathrm{BM}}$	0.20	Transmission Bird → Mosquito
$\beta_{\mathrm{MH}}$	0.05	Transmission Mosquito → Human
$\sigma_{B}$	0.33	Bird incubation rate
$\sigma_{M}$	0.20	Mosquito incubation rate
$\sigma_{H}$	0.25	Human incubation rate
$\gamma_{B}$	0.20	Bird recovery rate
$\gamma_{H}$	0.10	Human recovery rate
$\alpha_{B}$	0.02	Disease-induced mortality in birds
$\alpha_{M}$	0.01	Disease-induced mortality in mosquitoes
$\alpha_{H}$	0.00	Disease-induced mortality in humans
${ο}_{H}$	0.005	Waning immunity in humans

After the data preparation step, the proposed model was implemented in Python, taking advantage of several open-source libraries and frameworks that support network analysis, simulation, and modular experimentation. At the core of the implementation lies the NetworkX library, ^30^ which was used for the creation, manipulation, and analysis of graph structures. NetworkX provides a flexible and well-documented API that facilitated the representation of complex networks, as well as the computation of key topological properties required for both inference and evaluation.

To simulate network evolution and generate synthetic data reflecting realistic structural patterns, we employed the model introduced by Menczer and Fortunato, ^31^ in which offers a principled framework for modelling dynamic and heterogeneous networks. This simulation model allowed us to create controlled experimental conditions for assessing the robustness and generalizability of our method across different types of network topologies and growth dynamics.

The overall architecture and experimentation pipeline were structured using the ExDiff framework, an extensible platform designed for differential and explainable network inference. ^32^ ExDiff provided a modular environment for integrating multiple components, including data preprocessing, inference algorithms, and anomaly detection strategies, while enabling comparative benchmarking under consistent experimental protocols. Its plug-and-play design was essential for evaluating different algorithmic combinations and integration strategies within a unified framework.

All simulations and experiments were conducted using the Google Colab platform ( https://colab.research.google.com/), which offered a scalable and reproducible computational environment equipped with GPU acceleration and cloud-based resources. The use of Google Colab also facilitated collaboration and rapid prototyping, particularly during iterative development and evaluation phases.

The model was evaluated according to a multi-step protocol designed to assess both detection performance and integration efficiency. Simulation was carried out by adoping a network with 1,000 nodes, and a Stochastic Block Model Structure, in which we modelled three communities (Birds, Mosquitos and Human), with a probability of contact within the community p _1_=0.8 and a probability of contacts between the communities p _2_=0.2.

## West Nile diffusion model

WNV circulates within a complex ecological network mainly involving mosquitoes of the *Culex* genus and birds, which act as major reservoirs and amplifiers of the viral load. ^33^^,^^34^ The virus can spillover to incidental hosts, such as humans and horses, leading to a range of clinical manifestations from asymptomatic infection to severe neuroinvasive disease, ^35^ including meningitis and encephalitis. ^36^ Although these incidental hosts do not contribute substantially to transmission, the health impact of WNV episodes remains significant. Transmission dynamics are driven by a confluence of environmental and ecological factors. Mosquito abundance-one of the strongest predictors of WNV risk-is influenced by temperature and rainfall patterns that directly modulate vector capacity and the rate of viral replication within the vector. ^37^ In addition, the seasonal migration of birds influences the spatial and temporal availability of susceptible reservoir hosts, creating transient hotspots of viral amplification. These ecological variables, combined with human behaviour and urbanisation patterns, shape the landscape of WNV transmission and contribute to its spatial and temporal heterogeneity. Effective management of WNV requires an integrated understanding of the interactions between arthropod vectors, avian reservoirs and environmental modulators of risk. ^38, 39^ Multidisciplinary approaches combining entomological surveillance, ecological modelling and computational simulations are therefore essential to anticipate outbreak trajectories and design tailored vector control strategies. As no human vaccine currently exists, interventions must focus on suppressing mosquito populations and disrupting vector-host-outbreak contact chains that critically depend on the predictive insights offered by dynamic models. To capture the epidemiological dynamics of WNV, we adopt an extended SEIRD (Susceptible-Exposed-Infectious-Recovered-Dead) ^40, 41^ compartmental model that incorporates multiple host populations-birds, mosquitoes and humans-each with distinct biological and epidemiological roles.

**Figure 2.**
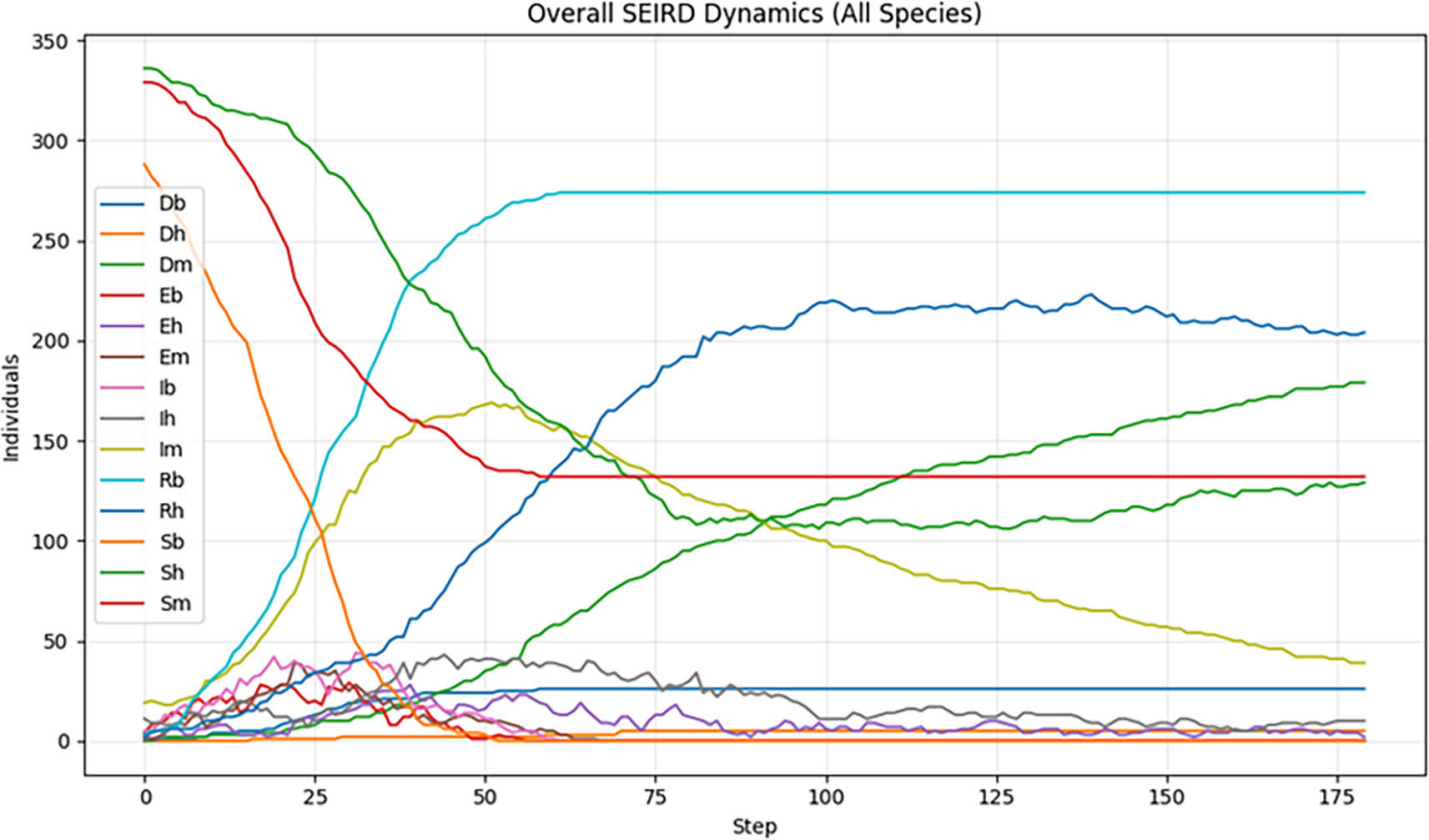
Overall SEIRD dynamics of WNV transmission across birds, mosquitoes, and humans using SEIRD baseline simulation over a 180‑day (≈6‑month) simulation period.

The interaction between these populations is encoded within a heterogeneous contact graph that is dynamic in time. A graphical representation of the structure of the model is shown in Figure 2. Birds act as the main amplifying hosts for WNV, and their dynamics are described via the compartments: *S*_*B*_ (Susceptible) for birds at risk of infection, $E_{B}$ (Exposed) for birds infected by a mosquito but not yet infectious, $I_{B}$ (Infectious) for birds capable of transmitting the virus to mosquitoes, $R_{B}$ (Recovered) for birds that have recovered and acquired immunity, $D_{B}$ (Dead) for birds that succumb to WNV infection. Mosquitoes, which act as vectors of transmission between birds and humans, do not recover from infection, but their life cycle includes mortality from both natural causes and control strategies: $S_{M}$ (Susceptible) for mosquitoes that have not yet acquired the virus, $E_{M}$ (Exposed) for mosquitoes that have bitten an infected bird but are still in the extrinsic incubation period, $I_{M}$ (Infectious) for mosquitoes capable of transmitting WNV, $D_{M}$ (Dead) for mosquitoes that die from natural causes, infection or interventions such as the use of larvicides or adulticides. Humans are considered incidental, end-of-cycle hosts, meaning that they do not contribute significantly to transmission. However, modelling morbidity and mortality is essential to capture health outcomes: $S_{H}$ (Susceptible) for individuals vulnerable to WNV infection, $E_{H}$ (Exposed) for individuals bitten by an infected mosquito and incubating the virus, $I_{H}$ (Infectious) for symptomatic individuals, an essential compartment for tracking the disease burden, $R_{H}$ (Recovered) for individuals who survive infection and acquire immunity, $D_{H}$ (Dead) for individuals who die due to WNV-related complications such as encephalitis or neuroinvasive disease.

SEIRD dynamics are interconnected through a graph-based interaction scheme reflecting biological transmission pathways: the Mosquito-Bird arcs represent the central zoonotic cycle for WNV amplification, while the Mosquito-Human arcs model the incidental spillover from the enzootic cycle to humans. There are no direct transmission arcs between humans or between birds, all transmission is vector-mediated. This modelling strategy enables high-resolution simulation of outbreak dynamics and evaluation of vector control interventions (e.g. mosquito population reduction), which is currently the only effective containment strategy in the absence of a human or avian vaccine. Figure 1 illustrates the multi-host SEIRD model and the direct edges encoding contact-based interactions that are critical for WNV transmission and control.

## Mathematical Equations of WNV

To provide further details on the interactions between the three epidemiological sub-models that drive the compartmental structure, as illustrated in Figure 1, this subsection presents the mathematical equations governing WNV diffusion. Each sub-model is linked to the next by the infection rate term, which is directly proportional to the infected fraction of the species capable of infecting the considered species.

Additionally, some simplifications were made:

• natural mortality rates were not considered
• newborns or new immigrants were not included
• mosquitoes can infect birds and vice versa, but humans cannot infect mosquitoes or birds
• no specific species of each organism were included.

Moreover, the simulations performed with this model are discrete events, where time evolves according to event occurrences rather than fixed intervals, thus the simulated time steps adapt dynamically based on transmission or recovery events. Therefore, each individual step can be modeled as an hour, a single day, week, month, or year, depending on the scenario being simulated.

*BIRDS*

$$\frac{{\mathrm{dS}}_{B}}{\mathrm{dt}}=-\lambda_{B}S_{B}$$

$$\frac{{\mathrm{dE}}_{B}}{\mathrm{dt}}=\lambda_{B}S_{B}-\sigma_{B}E_{B}$$

$$\frac{{\mathrm{dI}}_{B}}{\mathrm{dt}}=\sigma_{B}E_{B}-\gamma_{B}I_{B}-\alpha_{B}I_{B}$$

$$\frac{{\mathrm{dR}}_{B}}{\mathrm{dt}}=\gamma_{B}I_{B}$$

$$\frac{{\mathrm{dD}}_{B}}{\mathrm{dt}}=\alpha_{B}I_{B}$$

*where* $\lambda_{B}=\beta_{\mathrm{MB}}\frac{I_{M}}{N_{M}}\;$ is the infection rate for birds who can be infected by mosquitoes, $\sigma_{B}$ is the the inverse of the latency period, $\gamma_{B}$ is the recovery rate, $\alpha_{B}$ is the mortality rate due to the WNV for birds.

*MOSQUITOES*

$$\frac{{\mathrm{dS}}_{M}}{\mathrm{dt}}=-\lambda_{M}S_{M}-\mu_{M}S_{M}$$

$$\frac{{\mathrm{dE}}_{M}}{\mathrm{dt}}=\lambda_{M}S_{M}-\sigma_{M}E_{M}$$

$$\frac{{\mathrm{dI}}_{M}}{\mathrm{dt}}=\sigma_{M}E_{M}-\alpha_{M}I_{M}$$

$$\frac{{\mathrm{dD}}_{M}}{\mathrm{dt}}=\alpha_{M}I_{M}$$

*where* $\lambda_{M}=\beta_{\mathrm{BM}}\frac{I_{B}}{N_{B}}$ is the infection rate for mosquitoes who can be infected by birds, $\sigma_{M}$ is the the inverse of the latency period, $\gamma_{M}$ is the recovery rate, $\alpha_{M}$ is the mortality rate due to the WNV for mosquitoes.

*HUMANS*

$$\frac{{\mathrm{dS}}_{H}}{\mathrm{dt}}=-\lambda_{H}S_{H}+{ο}_{H}R_{H}$$

*where*

$$\lambda_{H}=\beta_{\mathrm{MH}}\frac{I_{M}}{N_{M}}\;\left(\text{infection rate mosquitoes influenced}\right)$$

$$\frac{{\mathrm{dE}}_{H}}{\mathrm{dt}}=\lambda_{H}S_{H}-\sigma_{H}E_{H}$$

$$\frac{{\mathrm{dI}}_{H}}{\mathrm{dt}}=\sigma_{H}E_{H}-\gamma_{H}I_{H}-\alpha_{H}I_{H}$$

$$\frac{{\mathrm{dR}}_{H}}{\mathrm{dt}}=\gamma_{H}I_{H}-{ο}_{H}R_{H}$$

$$\frac{{\mathrm{dD}}_{H}}{\mathrm{dt}}=\alpha_{H}I_{H}$$

*where* $\lambda_{H}=\beta_{\mathrm{MH}}\frac{I_{M}}{N_{M}}\;$ is the infection rate for mosquitoes who can be infected by birds, $\sigma_{H}$ is the the inverse of the latency period, ${ο}_{H}\;$ is the transition rate from recovered to susceptible, $\gamma_{H}$ is the recovery rate, $\alpha_{H}$ is the mortality rate due to the WNV for humans.

## Results

### Case Study 1: Uncontrolled Diffusion

Our baseline scenario simulates an uncontrolled WNV outbreak within a densely interconnected ecological network, with no containment intervention. This simulation establishes the basic conditions for the outbreak, characterised by high densities of mosquito vectors and a large population of susceptible birds-factors that favour continuous viral amplification and virus transmission. Human exposure occurs mainly through contact with infected mosquitoes that act as a transmission bridge. In the absence of vector control measures or environmental management, the simulation follows the natural propagation of the virus, which is governed solely by basic biological parameters: the competence of the vector, the incubation periods of the pathogen and the feeding behaviour of mosquitoes.

The basic results, summarized in Figure 2, reveal a rapid and extensive spread of WNV throughout the ecological network. During the early phases, infections rise sharply among both mosquito and bird populations, reflecting efficient amplification within avian hosts and the high turnover of mosquito vectors. The high degree of connectivity among bird populations, particularly migratory species that bridge geographically distant communities, enables efficient long-range dissemination of the virus. As the simulation progresses, the fraction of infected birds ( *I*_*B*_) stabilizes at elevated level maintaining a persistent reservoir of infection. In mosquitoes, the infected compartment ( *I*_*M*_) peaks early and then declines steadily, consistent with their short lifespan and limited capacity to sustain long transmission chains. Despite this decline, mosquitoes remain essential for cross-species transmission.

Human infections increase gradually but significantly over the 180-day period, driven by the interaction of persistent avian reservoirs and mosquito vectors. Although the absolute number of infected humans remains lower than in birds or mosquitoes, the upward trajectory highlights the substantial spillover risk in the absence of interventions. Without ecological interventions, such as larvicide application, adult mosquito suppression, or systematic monitoring of bird populations, the epidemic approaches critical transmission thresholds, saturating network pathways and resulting in a substantial cumulative burden of infection in the human population.

These baseline dynamics highlight the serious public health consequences of passive or delayed WNV response protocols. Natural transmission dynamics, when enhanced by favourable environmental conditions, can rapidly exceed the capacity of the local health system in the absence of timely and geographically targeted interventions. This scenario establishes the essential benchmarks for the evaluation of subsequent simulations, which incorporate active containment strategies, allowing for a quantitative assessment of the effectiveness of interventions, either through the suppression of mosquito populations or through the interruption of transmission cycles between birds and vectors.

### Case Study 2: Simulation of vector control intervention targeting mosquitoes

The containment scenario evaluates the impact of explainability-guided interventions applied to the GNN model of WNV transmission. We simulate this strategy by removing significant proportions of mosquito transmission links from the contact network, thereby cutting transmission paths between vectors and hosts. This approach simulates comprehensive control programmes, including large-scale larvicide deployment, adulticide spraying campaigns and targeted habitat eradication initiatives. By maintaining the integrity of bird and human population networks, but isolating the vector component, we can directly quantify the effects of mosquito suppression on epidemic trajectories.

**Figure 3.**
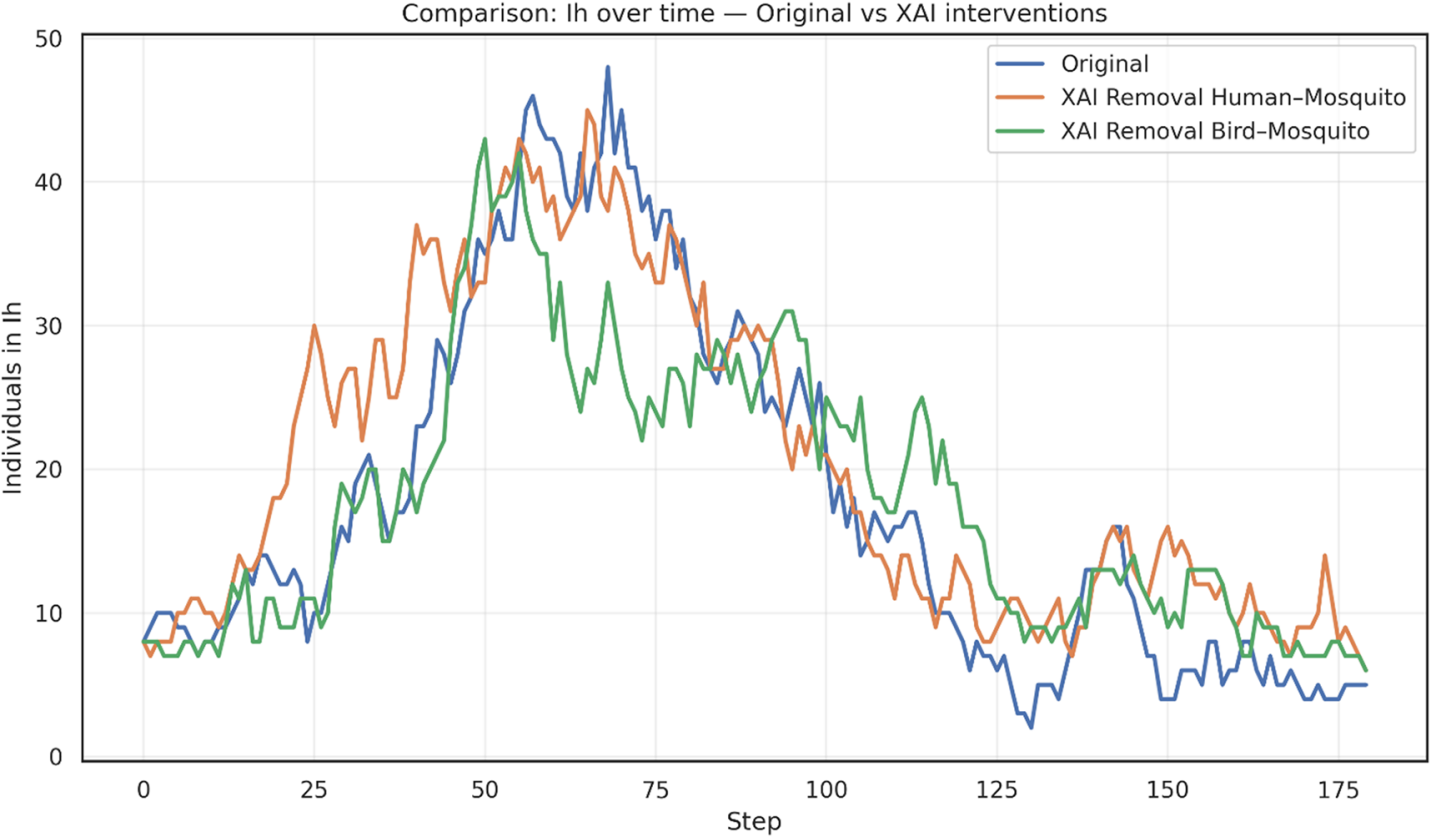
Temporal dynamics of infected humans ( *I*_*H*_) under the original model and XAI-based GNN interventions for WNV containment. Interventions consist of removing a percentage of highly important edges, as identified by explainability analysis, between species communities. The removal of human-mosquito connections leads to an earlier and sharper infection peak, whereas the removal of bird-mosquito connections results in a more moderate peak compared to the original scenario.

The results, shown in Figure 3, provide a conceptual framework for identifying and prioritizing critical transmission bridges. When human-mosquito edges are disrupted, the epidemic peak in infected humans ( *I*_*H*_) occurs earlier, but reaches a lower magnitude compared to the original scenario, suggesting that this pathway plays a central role in amplifying spillover risk. In contrast, removing bird-mosquito edges produces a more moderate peak of human infections, indicating that avian–vector interactions sustain transmission but with less immediate amplification in the human compartment.

In practical terms, the removal of human-mosquito edges can be interpreted as interventions that reduce direct exposure of people to mosquito bites (e.g., repellents, bed nets, protective barriers), whereas the removal of bird-mosquito edges reflects ecological or environmental measures aimed at limiting vector–reservoir interactions (e.g., habitat management, avifauna control, or monitoring of bird populations). These abstractions allow the XAI-based GNN framework to highlight how different classes of interventions may alter epidemic trajectories.

These results, shown in Figure 3, provides a conceptual framework for identifying and prioritizing critical transmission bridges. The high degree of connectivity among bird populations, particularly migratory species that link geographically distant communities, enables efficient long-range dissemination of the virus. This structural feature acts as a powerful driver of ecological amplification, sustaining viral circulation across spatially dispersed regions. The guided interventions, particularly cutting the bird–mosquito edges, reduces the amplification potential of avian reservoirs, thereby altering epidemic trajectories in ways that differ from interventions targeting human-mosquito interactions.

On the other hand, cutting the human–mosquito edges conceptually illustrates the central role of direct vector–human interactions in sustaining epidemic intensity. While not representing operational mosquito eradication, this abstraction highlights how interventions that reduce human exposure to mosquito bites can substantially alter outbreak trajectories, reinforcing the importance of prioritizing spillover prevention in WNV containment strategies.

Overall, the simulation highlights the value of explainability-driven approaches in distinguishing between direct spillover prevention and reservoir-vector disruption. Rather than providing evidence of operational mosquito eradication, these results emphasize how XAI-guided edge removal can conceptually inform the prioritization of containment strategies.

## Discussion

The results of this study strongly highlight the potential of network-based computational models to address the spread of WNV under realistic and complex scenarios. Unlike static approaches, the proposed framework enables dynamic simulation of the interactions among reservoir hosts, vectors, and human populations, incorporating targeted ecological interventions such as selective mosquito suppression or strategic management of bird populations. In particular, the integration of explainability-guided GNNs allows us to identify and selectively remove highly important transmission edges, providing a conceptual abstraction of interventions that either reduce human exposure to mosquito bites (human-mosquito edges) or limit vector-reservoir interactions (bird-mosquito edges). These capabilities are crucial in a context where no approved human vaccine is available, and public health responses must rely on non-pharmaceutical interventions. The two simulated scenarios clearly demonstrate the effectiveness of containment strategies. In the uncontrolled outbreak scenario, the epidemic spreads rapidly through the ecological network, with a surge in infected birds and mosquitoes and a significant increase in human cases. High connectivity among avian populations, especially migratory species, facilitates long-range viral transmission, illustrating how even minimal delays in intervention can lead to saturation of transmission pathways. This structural feature underscores the amplification potential of avian reservoirs, which can be conceptually mitigated in the model by cutting bird-mosquito edges. In contrast, the scenario involving targeted vector suppression shows a substantial reduction in transmission, ultimately breaking the epidemiological chain. Here, the removal of human–mosquito edges highlights the central role of direct vector-human interactions in sustaining epidemic intensity, conceptually illustrating how protective measures at the human-vector interface can alter outbreak trajectories. The drastic decrease in mosquito density reduces the number of spillover events to humans and prevents the virus from reaching a sustainable transmission level. These findings emphasize the importance of timely, geographically coordinated strategies for WNV control, confirming that well-implemented vector control measures remain one of the most effective tools for responding to vector-borne diseases. At the same time, the XAI-based framework demonstrates its added value by distinguishing between interventions that act directly on spillover risk and those that act upstream on ecological amplification.

Compared with previous WNV models, our framework advances the field by integrating explainability-guided GNNs with epidemic dynamics, enabling the identification of critical transmission edges and offering insights into the relative importance of distinct ecological pathways.

As detailed in the Mathematical Equations of WNV subsection, this work has some limitations due to the simplifications made in the mathematical model. One significant limitation is that we did not model multiple bird and mosquito species. Developing this aspect in future research would be valuable for studying its impact on transmission dynamics. Future extensions could also explore partial or regionally heterogeneous edge-removal strategies, reflecting more realistic intervention settings. Therefore, this paper effectively demonstrates how a novel, explainable AI framework can uniquely combine compartmental epidemic modelling with GNNs and interpretability to understand and manage the containment measures of viruses like MNV.

Beyond the methodological and epidemiological insights, the proposed framework also has potential operational value for public health agencies. By integrating network-based simulations and explainability-guided prioritization of transmission pathways, the tool could complement existing surveillance infrastructures, such as the Italian national arbovirus platform ArboItaly, by providing scenario-based risk assessments and supporting the timely allocation of vector-control resources. Its ability to dynamically incorporate ecological, entomological, and human mobility data makes it suitable for informing early-warning systems, guiding targeted interventions in high-risk areas, and enhancing preparedness strategies in regions where WNV emergence and re-emergence remain recurrent challenges.

## Data Availability

The static version of the dataset is deposited in Zenodo and accessible at https://zenodo.org/records/8355821. ^42^ To facilitate data reuse and ensure continuous updates, we also provide metadata, R scripts, and a dynamically maintained dataset in a dedicated GitHub repository: https://github.com/fbranda/west-nile .
