# Supplementary material for: Graph-based epidemic modeling of West Nile Virus: Forecasting and containment
Source: F1000Res. 2025 Dec 19;14:902. Originally published 2025 Sep 10. [Version 3] doi: 10.12688/f1000research.169601.3 (PMC12640483; doi:10.12688/f1000research.169601.3)
Supplement: Peer review history

# Reviewer report: Reviewer response for version 2

**DOI:** 10.5256/f1000research.190502.r432695

**Published:** 2025-11-22

**Authors:** Michael Wimberly

**Affiliations:** 1 The University of Oklahoma, Norman, Oklahoma, USA

The authors have made substantial modifications in response to the reviewer comments, and the readability of the paper, the interpretability of the results, and the relevance of the work to the broader field of disease ecology and modeling are all greatly enhanced as a result.

Is the rationale for developing the new method (or application) clearly explained?

No

Is the description of the method technically sound?

No

Are the conclusions about the method and its performance adequately supported by the findings presented in the article?

No

If any results are presented, are all the source data underlying the results available to ensure full reproducibility?

No

Are sufficient details provided to allow replication of the method development and its use by others?

No

Reviewer Expertise:

Disease ecology and modeling

I confirm that I have read this submission and believe that I have an appropriate level of expertise to confirm that it is of an acceptable scientific standard.

# Reviewer report: Reviewer response for version 1

**DOI:** 10.5256/f1000research.186955.r423796

**Published:** 2025-11-24

**Authors:** Katrin Gaardbo Kuhn, Gargi Deshpande

**Affiliations:** 1 The University of Oklahoma Health Sciences Center, Oklahoma City, Oklahoma, USA; 2 Biostatistics and Epidemiology, The University of Oklahoma Health Sciences Center, Oklahoma City, Oklahoma, USA

**Graph-based epidemic modeling of West Nile Virus: Forecasting and containment**

 The manuscript proposes a simulation framework for WNV transmission and the impact of prevention strategies on transmission dynamics using a network-based SEIRD model. The proposed model efficiently incorporates the different hosts and vectors of WNV- birds, mosquitoes, and humans, accounting for the complex interactions of these factors determining the transmission. The manuscript highlights that preventive strategies that focus on eliminating the infected mosquitoes can prevent further transmission to humans and prevent spillover events. 

 In general, the manuscript is timely and relevant, addressing an important gap in modeling vector-borne diseases under One Health principles. The conceptual foundation is strong, but I think that the technical and methodological details require clarification and expansion to make the model reproducible and its conclusions more robust. 

**Recommendation:** Major revisions required 

**General Comments: **

- I commend the authors for constructing a true One Health modeling approach; this is a much-needed step to better understand the transmission of zoonotic diseases. Having said that, I think there are major concerns with the construction of the model – primarily because the key parameters are not defined or listed anywhere in the manuscript. Where were the parameters obtained from and how have they been validated? 

- The temporal resolution of the model simulations is not clear, and this is an essential outcome for a seasonal disease like WNV. 

- The description of Case study 1 and 2 are well written, but both lack defining the assumptions that are made in these models. Assumptions such as vector’s ability to acquire, maintain and transmit the virus or host preference and biting rate, effectiveness of control measures should be outlined. 

- In Case study 2, eliminating all mosquitoes from the network seems to be a simplistic approach. The model may benefit by conducting a sensitivity analysis where you gradually eliminate the mosquitoes and see its impact on the infected mosquito population and human infections. Results generated for varying levels of mosquito elimination will also account for the heterogeneity in the effectiveness of mosquito elimination strategies, while also providing a comprehensive result for human infections. 

- For the vector control component, the description of interventions is very vague and there is no description of how these interventions correspond to real-life control measures. Also, to which extent is the approach scalable? 

- WNV vectors bite different species of birds, and this is not accounted for in the model. At the very least, the multi-species bird compartment issue should be emphasized in the discussion (provided that it is not possible to incorporate it with parameters). 

- The discussion section would benefit from stronger integration with the existing literature. Currently, it lacks sufficient referencing and does not adequately position the proposed model in context of previous studies that have developed similar simulation frameworks for WNV or other mosquito-borne diseases. This section also doesn’t discuss any limitations or adaptability of the framework. It would be useful to discuss how this model could be extended, calibrated, or customized to support targeted vector control strategies and prevent spillover events in different ecological and geographical contexts. 

**Minor Comments: **

- Some sentences are overly complex and could be split for clarity (especially in the Introduction and Methods sections). 
- Figures:  
- In figure 1 label each of the three epidemiological submodels-Birds, mosquitoes and humans to make it easier to understand. Also, improve readability (labels are small). 
- In figure 1 the description of the figure says- “Specifically, mosquitoes acquire infection from infectious birds (I _b_) and transmit the virus to susceptible humans (S _h_)”, but none of the dotted arrows point to susceptible humans. The transmission route shown in the figure is, infected bird>exposed mosquito>infected mosquito>exposed human which can be misleading based on the description. 
- Harmonize color schemes between Figures 2 and 3. 
- The materials and methods section states- “Simulation was carried out by adopting a network with 1,000 nodes, and a Stochastic Block Model Structure, in which we modelled three communities (Birds, Mosquitos and Human), with a probability of contact within the community p1=0.8 and a probability of contacts between the communities p2=0.2.” Outlining the rationale or providing a reference on why these probabilities were selected would be useful. 

- In the results section, it is stated- “The drastic reduction of vector density brings the basic reproduction number below the critical threshold of 1, effectively interrupting self-sustaining transmission cycles.” Instead of using drastic, quantify the result to increase its interpretability. 

- The introduction states- “Using simulation scenarios, we explore the timing, localization, and intensity of mosquito population suppression and its effect on outbreak size, duration, and mortality.” However, the study does not explore aspects like timing, localization and intensity in their analysis. Please revise this statement to more accurately reflect the scope and outcomes of the study. 

- Add a short paragraph in the Discussion linking the proposed tool to potential policy or operational uses (e.g., integration with public health surveillance platforms like ArboItaly). 

Is the rationale for developing the new method (or application) clearly explained?

Partly

Is the description of the method technically sound?

Yes

Are the conclusions about the method and its performance adequately supported by the findings presented in the article?

Partly

If any results are presented, are all the source data underlying the results available to ensure full reproducibility?

No

Are sufficient details provided to allow replication of the method development and its use by others?

No

Reviewer Expertise:

Vector-borne diseases. environmental epidemiology, One Health, infectious diseases epidemiology, climate change, zoonotic diseases.

We confirm that we have read this submission and believe that we have an appropriate level of expertise to confirm that it is of an acceptable scientific standard, however we have significant reservations, as outlined above.

# Response

**Published:** 2025-12-06

**Authors:** Mohamed Mustaf Ahmed

**Affiliations:** Medicine and Health Sciences, SIMAD University, Mogadishu, Banaadir, Somalia

**General Comments:**

 1. I commend the authors for constructing a true One Health modeling approach; this is a much-needed step to better understand the transmission of zoonotic diseases. Having said that, I think there are major concerns with the construction of the model – primarily because the key parameters are not defined or listed anywhere in the manuscript. Where were the parameters obtained from and how have they been validated? 

**We appreciate the reviewer’s positive assessment of our effort to develop a genuine One Health modeling framework. In the revised version of the manuscript, we have now addressed the concerns regarding parameter specification and validation. Specifically, we have added a complete list of all key model parameters, clarified their definitions, and detailed their sources (literature, surveillance data, or expert elicitation). We also included a dedicated subsection describing the procedures used to validate these parameters—both through comparison with published estimates and through internal model calibration. These additions ensure that the model’s construction is fully transparent and reproducible.**

 2. The temporal resolution of the model simulations is not clear, and this is an essential outcome for a seasonal disease like WNV.

**We thank the reviewer for highlighting the importance of temporal resolution, particularly for a seasonal disease such as WNV. In version 2 of the manuscript, we have clarified the temporal resolution of all model simulations. We now explicitly describe the time step used in the model, how seasonal forcing is incorporated, and how these choices align with the epidemiological dynamics of WNV. These details have been added to the Methods section to ensure full transparency and reproducibility.**

 3. The description of Case study 1 and 2 are well written, but both lack defining the assumptions that are made in these models. Assumptions such as vector’s ability to acquire, maintain and transmit the virus or host preference and biting rate, effectiveness of control measures should be outlined. 

**The primary contribution of this paper is to demonstrate the superior applicability of a novel explainable AI framework designed to foster new research directions in vector-borne disease modelling.**

 4. In Case study 2, eliminating all mosquitoes from the network seems to be a simplistic approach. The model may benefit by conducting a sensitivity analysis where you gradually eliminate the mosquitoes and see its impact on the infected mosquito population and human infections. Results generated for varying levels of mosquito elimination will also account for the heterogeneity in the effectiveness of mosquito elimination strategies, while also providing a comprehensive result for human infections.

**V2: We simulate this strategy by removing significant proportions of mosquito nodes and their transmission links from the contact network, thereby cutting transmission paths between vectors and hosts.**

 5. For the vector control component, the description of interventions is very vague and there is no description of how these interventions correspond to real-life control measures. Also, to which extent is the approach scalable?

**The approach is scalable, as the procedure can be applied to networks of progressively larger size, while allowing the user to adjust the extent of edge removal in accordance with the requirements of the specific application.”**

 6. WNV vectors bite different species of birds, and this is not accounted for in the model. At the very least, the multi-species bird compartment issue should be emphasized in the discussion (provided that it is not possible to incorporate it with parameters). 

**V2: One significant limitation is that we did not model multiple bird and mosquito species. Developing this aspect in future research would be valuable for studying its impact on transmission dynamics.**

**We acknowledge that one significant limitation is that we did not model multiple bird and mosquito species. Developing this aspect in future research would be valuable for studying its impact on transmission dynamics. Therefore, the primary contribution of this paper is to demonstrate the superior applicability of a novel explainable AI framework designed to foster new research directions in vector-borne disease modelling.**

 7. The discussion section would benefit from stronger integration with the existing literature. Currently, it lacks sufficient referencing and does not adequately position the proposed model in context of previous studies that have developed similar simulation frameworks for WNV or other mosquito-borne diseases. This section also doesn’t discuss any limitations or adaptability of the framework. It would be useful to discuss how this model could be extended, calibrated, or customized to support targeted vector control strategies and prevent spillover events in different ecological and geographical contexts. 

**We improved both related work and discussion.**

**Minor Comments:**

 1. Some sentences are overly complex and could be split for clarity (especially in the Introduction and Methods sections). 

**We checked and polished the manuscript.**

 2. Figures:  

-
  - In figure 1 label each of the three epidemiological submodels-Birds, mosquitoes and humans to make it easier to understand. Also, improve readability (labels are small). 
  - In figure 1 the description of the figure says- “Specifically, mosquitoes acquire infection from infectious birds (Ib) and transmit the virus to susceptible humans (Sh)”, but none of the dotted arrows point to susceptible humans. The transmission route shown in the figure is, infected bird>exposed mosquito>infected mosquito>exposed human which can be misleading based on the description. 
  - Harmonize color schemes between Figures 2 and 3. 

**  **


**We improved the quality of all the figures.**

 3. The materials and methods section states- “Simulation was carried out by adopting a network with 1,000 nodes, and a Stochastic Block Model Structure, in which we modelled three communities (Birds, Mosquitos and Human), with a probability of contact within the community p1=0.8 and a probability of contacts between the communities p2=0.2.” Outlining the rationale or providing a reference on why these probabilities were selected would be useful.

**The choice of probabilities p1=0.8 (within-community) and p2=0.2 (between-community)  was motivated by the need to generate a clear modular structure in the Stochastic Block Model, which reflects the expected higher frequency of contacts within species compared to cross-species interactions. These values were selected for illustrative purposes, to ensure a detectable community structure in the simulations.**

 4. In the results section, it is stated- “The drastic reduction of vector density brings the basic reproduction number below the critical threshold of 1, effectively interrupting self-sustaining transmission cycles.” Instead of using drastic, quantify the result to increase its interpretability.

**We detailed the result section to discuss these details.**

 5. The introduction states- “Using simulation scenarios, we explore the timing, localization, and intensity of mosquito population suppression and its effect on outbreak size, duration, and mortality.” However, the study does not explore aspects like timing, localization and intensity in their analysis. Please revise this statement to more accurately reflect the scope and outcomes of the study.

**We deleted localization word.**

 6. Add a short paragraph in the Discussion linking the proposed tool to potential policy or operational uses (e.g., integration with public health surveillance platforms like ArboItaly).

**We improved the discussion.**

# Reviewer report: Reviewer response for version 1

**DOI:** 10.5256/f1000research.186955.r414393

**Published:** 2025-10-25

**Authors:** Laura Antonelli

**Affiliations:** 1 Istituto of High Performance Computing and Networks, National Research Council, Via P. Castellino, Naples, Italy

This study provides a substantial conceptual and methodological advancement in modelling West Nile Virus (WNV) transmission through an adaptive, network-based SEIRD framework. By situating WNV within the broader context of global infectious disease modelling, the paper articulates a well-founded rationale for the adoption of computational approaches, particularly in scenarios where vaccine-based interventions are unavailable. The focus on network-based representations, capable of capturing the complex and heterogeneous ecological interactions among mosquitoes, avian reservoirs, and  human hosts, is both scientifically rigorous and conceptually sophisticated. Furthermore, the proposed framework effectively integrates principles from epidemiology, data science, and ecology, thereby embodying a comprehensive and multidisciplinary “One Health” approach.

 In my opinion, the manuscript requires minor corrections as suggested below:

- Some sentences are too long and syntactically complex, which can obscure meaning (e.g., “The interaction between these populations is encoded within a heterogeneous contact graph that is dynamic in time…” could be simplified). Enhance readability and flow by using shorter sentences and clearer structural divisions. 
- Consider a brief comparative paragraph discussing how this model advances or differs from prior WNV modelling studies.
- Citations are frequent and well-placed, but are inconsistently formatted (some are numeric references, others have textual mentions); you should use a standard format.
- Some characters in the figures are unreadable (e.g. see Fig. 1, the second blue ball)
- The titles of Figures 2 and 3 are redundant and could be removed

Is the rationale for developing the new method (or application) clearly explained?

Yes

Is the description of the method technically sound?

Yes

Are the conclusions about the method and its performance adequately supported by the findings presented in the article?

Yes

If any results are presented, are all the source data underlying the results available to ensure full reproducibility?

Yes

Are sufficient details provided to allow replication of the method development and its use by others?

Yes

Reviewer Expertise:

Computational Modelling & Scientific Computing for AI

I confirm that I have read this submission and believe that I have an appropriate level of expertise to confirm that it is of an acceptable scientific standard.

# Response

**Published:** 2025-10-29

**Authors:** Mohamed Mustaf Ahmed

**Affiliations:** Medicine and Health Sciences, SIMAD University, Mogadishu, Banaadir, Somalia

GENERAL COMMENT: This study provides a substantial conceptual and methodological advancement in modelling West Nile Virus (WNV) transmission through an adaptive, network-based SEIRD framework. By situating WNV within the broader context of global infectious disease modelling, the paper articulates a well-founded rationale for the adoption of computational approaches, particularly in scenarios where vaccine-based interventions are unavailable. The focus on network-based representations, capable of capturing the complex and heterogeneous ecological interactions among mosquitoes, avian reservoirs, and  human hosts, is both scientifically rigorous and conceptually sophisticated. Furthermore, the proposed framework effectively integrates principles from epidemiology, data science, and ecology, thereby embodying a comprehensive and multidisciplinary “One Health” approach. n my opinion, the manuscript requires minor corrections as suggested below:

**Comment 1:** Some sentences are too long and syntactically complex, which can obscure meaning (e.g., “The interaction between these populations is encoded within a heterogeneous contact graph that is dynamic in time…” could be simplified). Enhance readability and flow by using shorter sentences and clearer structural divisions. 

**Response 1: **We appreciate the reviewer's suggestion and have revised the paper to enhance its structure and improve the flow of the discussion.

**Comment 2: **Consider a brief comparative paragraph discussing how this model advances or differs from prior WNV modelling studies. 

**Response 2: **We thank the reviewer for this feedback. We added a related work section to contextualize the study and we expanded the discussion highlighting this point.

**Comment 3: **Citations are frequent and well-placed, but are inconsistently formatted (some are numeric references, others have textual mentions); you should use a standard format.

**Response 3: **We appreciate the reviewer’s comment, and we fixed these formatting inconsistencies.

**Comment 4:** Some characters in the figures are unreadable (e.g. see Fig. 1, the second blue ball)

**Response 4: **We thank the reviewer for the constructive feedback. We have recreated Figure 1 to enhance its precision and readability.

**Comment 5:** The titles of Figures 2 and 3 are redundant and could be removed. 

**Response 5: **We thank the reviewer for this suggestion. We updated the figures and improved the captions, removing the redundancy of the titles.

# Reviewer report: Reviewer response for version 1

**DOI:** 10.5256/f1000research.186955.r417875

**Published:** 2025-10-15

**Authors:** Michael Wimberly

**Affiliations:** 1 The University of Oklahoma, Norman, Oklahoma, USA

The idea of simulating West Nile virus using a mechanistic compartment model is interesting and has the potential to yield new insights into the drivers of transmission and the effectiveness of various treatment strategies. However, the manuscript in its current form has a variety of issues that limit its impact.

 In general, the description of the model is not sufficient to be able to understand and evaluate the results. The graph in Figure 1 is helpful, but it is also very important to include the mathematical equations governing transitions between the compartments along with other details such as the length of the time step (hours, days, weeks?). I was also surprised to find that there were no details at all about model parameterization. Were parameters obtained from the literature? Or were they estimated through a model fitting process? In either case, much more information is needed about these procedures, and all the key parameters need to be provided in the paper.

 From what I can see in the paper, it looks like the model has only a single set of compartments representing birds. However, WNV vector mosquitoes bite multiple species of birds, and different species have different levels of vector competence. Thus, the species composition of the avian community combined with mosquito feeding preferences for different species can have a strong influence on transmission dynamics. Similarly, there is no discussion of the species of vector mosquitoes being modeled, but different species have very different life history parameters that can have a strong effect on transmission dynamics.

 The study highlights differences between two scenarios, one with mosquito control and one without.  It is unclear how the mosquito control intervention is represented in the model – the text describes “removing significant proportions of mosquito nodes and their transmission links”, but I cannot tell if this is a realistic representation of a vector control intervention. Unsurprisingly, this change results in reductions of infected mosquitoes, birds, and humans. Overall, the results are more a proof of concept demonstration than a real modeling experiment. This type of study should include a sensitivity analysis to provide understanding of the relative importance of various model parameters and should involve experiments that are designed to test elements of ecological theory or practical questions about the effectiveness of different disease control strategies.

 I would also note that this type of modeling approach is not unique. There are a number of published studies that have used this type of compartment-based models for WNV, as well as for other vector-borne diseases such as malaria and dengue. The paper should include a thorough review of the relevant scientific literature and should highlight how this model builds upon previous research.

 A minor comment on the figures – the same color scheme should be used for Figures 2 and 3 so they can be more easily compared.

Is the rationale for developing the new method (or application) clearly explained?

No

Is the description of the method technically sound?

No

Are the conclusions about the method and its performance adequately supported by the findings presented in the article?

No

If any results are presented, are all the source data underlying the results available to ensure full reproducibility?

No

Are sufficient details provided to allow replication of the method development and its use by others?

No

Reviewer Expertise:

Disease ecology and modeling

I confirm that I have read this submission and believe that I have an appropriate level of expertise to state that I do not consider it to be of an acceptable scientific standard, for reasons outlined above.

# Response

**Published:** 2025-10-29

**Authors:** Mohamed Mustaf Ahmed

**Affiliations:** Medicine and Health Sciences, SIMAD University, Mogadishu, Banaadir, Somalia

GENERAL COMMENT: The idea of simulating West Nile virus using a mechanistic compartment model is interesting and has the potential to yield new insights into the drivers of transmission and the effectiveness of various treatment strategies. However, the manuscript in its current form has a variety of issues that limit its impact.

**Comment 1: **In general, the description of the model is not sufficient to be able to understand and evaluate the results. The graph in Figure 1 is helpful, but it is also very important to include the mathematical equations governing transitions between the compartments along with other details such as the length of the time step (hours, days, weeks?). 

**Response 1: **We thank the reviewer for this important feedback. In response, we have enriched the manuscript by adding detailed mathematical formulations governing the compartment transitions in and around Figure 1, clearly specifying all state variables and equations. We also provide a short subsection providing additional details (Mathematical Equations of WNV) on the mathematical model. Moreover, we clarify that our simulation is a discrete event model, where time evolves according to event occurrences rather than fixed intervals, thus the simulated time steps adapt dynamically based on transmission or recovery events. Therefore, each individual step can be modeled as an hour, a single day, week, month, or year, depending on the scenario being simulated. 

**Comment 2: **I was also surprised to find that there were no details at all about model parameterization. Were parameters obtained from the literature? Or were they estimated through a model fitting process? In either case, much more information is needed about these procedures, and all the key parameters need to be provided in the paper. 

**Response 2: **We thank the reviewer for pointing out this aspect. Regarding parameterization, all key epidemiological and biological parameters used to describe the kinetic dynamics of WNV, including incubation periods, recovery times, disease-induced mortality, and inter-species transmission probabilities, were derived exclusively from a systematic review of the established literature on WNV epidemiology and modelling. These parameters are now explicitly listed in a dedicated section with corresponding references to ensure reproducibility and transparency. No crucial parameters related to viral spread were estimated through model fitting on Italian surveillance data. Regional case data were employed solely for contextual enrichment and qualitative validation of the simulated epidemic trajectories, ensuring that the scenarios reflect realistic outbreak dynamics. This methodology, grounded in literature-based parameterization, is essential to preserve the generalizability of the model. Incorporating these parameters into a network-based simulation framework allows the isolation and analysis of ecological and structural interactions, such as population connectivity, on the effectiveness of non-pharmaceutical interventions, without the baseline viral dynamics being distorted by local uncertainties or variability from statistical fitting. 

 Nevertheless, we emphasize that the primary aim of this paper is to show how a novel, explainable AI framework can uniquely combine compartmental epidemic modelling with Graph Neural Networks (GNNs) and interpretability. The platform enables other researchers to simulate outbreaks using their own data and parameter sets in a flexible, open manner, fostering broader use and further model refinement beyond fixed parameter scenarios. This balances mechanistic rigor with AI-driven adaptability, expanding possibilities for vector-borne disease modelling. 

**Comment 3: **From what I can see in the paper, it looks like the model has only a single set of compartments representing birds. However, WNV vector mosquitoes bite multiple species of birds, and different species have different levels of vector competence. Thus, the species composition of the avian community combined with mosquito feeding preferences for different species can have a strong influence on transmission dynamics. Similarly, there is no discussion of the species of vector mosquitoes being modeled, but different species have very different life history parameters that can have a strong effect on transmission dynamics. 

**Response 3: **We appreciate the reviewer’s thoughtful comment regarding  multiple species of organisms in our model. We acknowledge that one significant limitation is that we did not model multiple bird and mosquito species. Developing this aspect in future research would be valuable for studying its impact on transmission dynamics. Therefore, the primary contribution of this paper is to demonstrate the superior applicability of a novel explainable AI framework designed to foster new research directions in vector-borne disease modelling. 

**Comment 4: **The study highlights differences between two scenarios, one with mosquito control and one without.  It is unclear how the mosquito control intervention is represented in the model – the text describes “removing significant proportions of mosquito nodes and their transmission links”, but I cannot tell if this is a realistic representation of a vector control intervention. Unsurprisingly, this change results in reductions of infected mosquitoes, birds, and humans. 

**Response 4: **We thank the reviewer for these highlights. We detailed the proposed intervention strategies in the dedicated section.  Regarding the intervention scenarios, the simulated removal of highly important mosquito transmission edges corresponds to a stylized abstraction of targeted control strategies. In practical terms, the removal of human–mosquito edges reflects measures that reduce human exposure to mosquito bites (e.g., repellents, bed nets, protective barriers), while the removal of bird-mosquito edges represents ecological or environmental actions aimed at limiting vector-reservoir interactions (e.g., habitat management, avifauna control, population monitoring). Such interventions have been documented as key components of Italy and Europe’s public health response to West Nile Virus outbreaks and other mosquito-borne diseases. We stress that our model’s modular design allows users to modify these intervention parameters freely, enabling them to simulate partial, delayed, or regionally heterogeneous control efforts. This flexibility supports exploration of diverse and more nuanced control strategies aligned with local realities, while the present full-removal scenario serves as a conceptual benchmark demonstrating the potential impact of aggressive mosquito control. Overall, this framework aims to empower researchers and public health practitioners to test and adapt outbreak simulations with realistic or hypothetical intervention settings tailored to their specific use cases. 

**Comment 5: **Overall, the results are more a proof of concept demonstration than a real modeling experiment. This type of study should include a sensitivity analysis to provide understanding of the relative importance of various model parameters and should involve experiments that are designed to test elements of ecological theory or practical questions about the effectiveness of different disease control strategies.

**Response 5:** We appreciate the reviewer’s insightful comment and fully agree with this perspective. The primary focus of our study is to present a new explainable AI framework that combines mechanistic compartmental epidemic modelling with graph neural networks, demonstrating its usability and the reliability of produced results through proof-of-concept simulations. While our current work establishes the platform’s foundational capabilities, it does not yet delve deeply into detailed ecological theory testing or extensive practical disease control scenario evaluations such as sensitivity analyses.

 We acknowledge that sensitivity analysis is crucial for identifying influential parameters and understanding model behavior in infectious disease modelling. Such analyses are valuable for refining models and optimizing control strategies once the computational framework is mature. Our framework is designed to be extensible, enabling future users to incorporate these rigorous sensitivity and scenario testing workflows. Subsequent studies leveraging this platform can integrate global or local sensitivity analyses to assess parameter importance and test ecological or intervention hypotheses in vector-borne disease contexts. Thus, our work lays the methodological groundwork that encourages and facilitates these important next steps in research. 

**Comment 6:** I would also note that this type of modeling approach is not unique. There are a number of published studies that have used this type of compartment-based models for WNV, as well as for other vector-borne diseases such as malaria and dengue. The paper should include a thorough review of the relevant scientific literature and should highlight how this model builds upon previous research. 

**Response 6: **We appreciate the reviewer’s insightful comment on the need to situate our modelling approach within the context of prior research. In response, we have expanded the manuscript to include a Related Work section that discusses existing computational models for West Nile Virus (WNV) and other vector-borne diseases. Indeed, several studies have explored compartment- or network-based frameworks for WNV, including recent applications of Graph Neural Networks (GNNs) for disease forecasting. For example, Tonks et al. (2022, 2024) proposed spatially aware GNN models using GraphSAGE layers to predict WNV presence in Illinois based on mosquito surveillance data. Similarly, Bonicelli et al. (2023) applied GNN-based aggregation to model spatial circulation patterns of WNV , while semi-supervised GNN architectures have been explored for WNV prevalence forecasting using limited mosquito trap and environmental data. For dengue applications, recent GNN models with attention mechanisms have improved predictive accuracy and interpretability in disease severity prediction. However, none of these studies have integrated graph-based epidemic modelling with explicit mechanistic compartments and explainable inference, as presented in our work. Our framework is, to our knowledge, the first to couple compartmental disease dynamics (SEIRD structure) with graph neural network representation and explainability modules, enabling both mechanistic interpretability and data-driven learning within a unified system. This integration advances prior compartment models by introducing an explainable GNN architecture capable of identifying transmission pathways, offering novel insights into vector-host-human interactions and intervention effectiveness. This addition to the manuscript clarifies how our model not only builds upon but also significantly extends earlier GNN-based and compartmental approaches, establishing a methodological bridge between mechanistic epidemiology and explainable artificial intelligence. 

**Comment 7: **A minor comment on the figures – the same color scheme should be used for Figures 2 and 3 so they can be more easily compared.

**Response 7: ** We acknowledge that the previous version of the figures could cause misunderstanding due to the colours, even with an explicit legend. In the updated version of our work, the figures have been revised and improved for easier understanding.
